# Water, Water, Everywhere: Defining and Assessing Data Sharing in Academia

**DOI:** 10.1371/journal.pone.0147942

**Published:** 2016-02-17

**Authors:** Steven Van Tuyl, Amanda L. Whitmire

**Affiliations:** 1 Center for Digital Scholarship and Services, University Libraries, Oregon State University, Corvallis, Oregon, United States of America; 2 Harold A. Miller Library, Hopkins Marine Station, Stanford University, Pacific Grove, California, United States of America; Hellas, GREECE

## Abstract

Sharing of research data has begun to gain traction in many areas of the sciences in the past few years because of changing expectations from the scientific community, funding agencies, and academic journals. National Science Foundation (NSF) requirements for a data management plan (DMP) went into effect in 2011, with the intent of facilitating the dissemination and sharing of research results. Many projects that were funded during 2011 and 2012 should now have implemented the elements of the data management plans required for their grant proposals. In this paper we define ‘data sharing’ and present a protocol for assessing whether data have been shared and how effective the sharing was. We then evaluate the data sharing practices of researchers funded by the NSF at Oregon State University in two ways: by attempting to discover project-level research data using the associated DMP as a starting point, and by examining data sharing associated with journal articles that acknowledge NSF support. Sharing at both the project level and the journal article level was not carried out in the majority of cases, and when sharing was accomplished, the shared data were often of questionable usability due to access, documentation, and formatting issues. We close the article by offering recommendations for how data producers, journal publishers, data repositories, and funding agencies can facilitate the process of sharing data in a meaningful way.

## Introduction

*“It is one thing to encourage data deposition and resource sharing through guidelines and policy statements*, *and quite another to ensure that it happens in practice*.*”* [1]

In 2011, the National Science Foundation (NSF) reaffirmed a longstanding requirement for the dissemination and sharing of research results by adding a requirement for the submission of a data management plan (DMP) with grant proposals [2]. DMPs are intended to explain how researchers will address the requirement that they will “share with other researchers, at no more than incremental cost and within a reasonable time, the primary data, samples, physical collections and other supporting materials created or gathered in the course of work under NSF grants. Grantees are expected to encourage and facilitate such sharing” [3]. The expectation that NSF-funded researchers will share data has been in place since at least 1995, the year of the oldest NSF Grant Proposal Guide that we could locate in the NSF online archive [4], but the requirement is likely much older. A memorandum put forth by the White House Office of Science and Technology Policy (OSTP) in 2013 aimed at ensuring public access to the results of federally funded research [5], and the subsequent responses from funding agencies, lends credence to the notion that Federal funding agencies are now beginning to take seriously the idea that federally funded data are products that should be managed and shared in order to maximize scientific output from federal investments.

While the NSF does not currently require sharing the dataset that underlies an article at the time of publication, many scientific journals have begun to require or request data sharing as part of the publication process [6]. This move has been motivated by recent high profile cases of scientific misconduct related to falsified/poorly analyzed data [7] and the increasing acknowledgment among scientific communities that data sharing should be part of the process of communicating research results [8–11].

A challenge has arisen, though, of defining data sharing in a way that is useful to a broad spectrum of data producers and consumers. The NSF, for example, has been reluctant to define not only data sharing or data sharing best practices, but the meaning of data itself, insisting that these definitions should “be determined by the community of interest through the process of peer review and program management”, rather than being mandated [12]. This lack of guidance has caused some level of confusion among researchers trying to share data, and among service providers attempting to offer venues for data sharing. We have begun to see communities of practice offering guidance on best practices for data sharing from individual research domains (for examples see references [1,13,14] and DataONE.org) and from broad-level organizations such as Force11 [15] and Kratz and Strasser [9]. While many of these resources are helpful for understanding how to effectively share data, we have yet to see a rubric for evaluating how well a dataset is shared and assessing where improvements should be made to facilitate more effective sharing.

In this study we set a definition of data sharing and create a rubric for evaluating how well data have been shared at two significant levels: for research projects as a whole and as a dataset that underlies a journal article. We focus on research projects because of the NSF and OSTP focus on project-level requirements for data sharing (as cited above), and on journal articles because these represent a logical and common venue for data sharing [16]. We use our rubric to evaluate data sharing from NSF-funded projects that were put into effect after the requirements for management and sharing plan was put into place. Likewise, we use our rubric to evaluate data sharing in journal articles that originate from NSF-funded research projects that are subject to said policy. We conclude by offering guidance on best practices for facilitating data sharing to authors, journals, data repositories, and funding agencies.

## Methods

### Definition of Data Sharing

In this paper, we define criteria for assessing the effectiveness of data sharing under the assumption that the goal of data sharing is to make the data usable, with minimal effort, to the new user. We take the position that the bar should be set relatively low for the usability of data in order to facilitate the downstream goals of sharing such as validation of results, reproducibility of research, and use of data for novel science.

The criteria chosen for this research (elaborated below) were developed in consideration of recommendations from the academic literature (e.g. [9,13,15]), from best practices identified by organizations focused on data sharing (e.g. [15] and DataONE), and our experiences as data service providers and data users. Based on these sources, we define data sharing as addressing four criteria: Discoverability, Accessibility, Transparency, and Actionability (DATA). Specifically, data should be:

- Discoverable—one should be able to get to the data using direct and persistent links [13,15]. Pointing in a general way to a repository or database without providing an identifier or specific location does not ensure that the intended dataset will be found. Lack of specificity in location results in a lower discoverability score.
- Accessible—the data should be shared via an open platform with minimal barriers to access [9,13,15]. Examples of barriers to access would be having to contact the dataset creator for permission to use the dataset or having to provide an explanation for how or why one wants to use the data.
- Transparent—the data should have collocated documentation that describes it sufficiently for an expert to use [9,13,15]. Citing the methods section of an article is not sufficient because articles lack significant details that describe the dataset (definition of headers, units, processing steps, etc.). Relying on a paper for dataset description is also strongly discouraged because most papers are not accessible without a subscription to the parent journal. Likewise, referring to external community standards of practice (SOP) is not likely to be a robust descriptive mechanism over the long term as SOPs change over time and their provenance may not be clearly documented.
- Actionable—one should be able to use the data in analytical platforms with minimal reprocessing or reformatting [13,15]. For example, sharing quantitative data as a figure in an article or as a table in a PDF requires burdensome reformatting. These data are not considered actionable.

### NSF Funded Project Data and Protocol

We used an advanced search of the NSF Awards Database to identify NSF-funded projects at Oregon State University (OSU) that started on or after the start date of the DMP requirement (18 January 2011) through the end of 2013. Projects with a later start date than that are not likely to be far enough along to have much shared data. We set an end date parameter of 01 July 2015 in order to exclude ongoing projects that would overlap with this research. While we recognize that projects with a recent end date are not required to have shared any data yet, we wanted to avoid unnecessarily excluding projects that may have shared data during the course of the research. This query resulted in 91 projects. Within this set of search results, the OSU Office of Sponsored Programs was able to provide us with about one-third of the DMPs (N = 33).

The process of attempting to locate datasets based on a DMP started in the most obvious way: by looking where the DMP stated that data would be made available. If a dataset was not found in the location specified in the DMP, we looked in three additional locations (listed below). Given that it can be years between when a proposal is submitted and when a dataset is ready to be shared, we anticipated that there would be deviations from DMPs in the actual venue for data sharing. Our search protocol for discovering datasets associated with these DMPs therefore included looking in the following places, in order:

1. Location specified in DMP, if applicable
2. NSF award page (use a simple search; include expired awards)
3. PI website(s), research group or project website
4. DataCite Metadata Search for author name (http://search.datacite.org/ui)

We used the DataCite Metadata Search as a catch-all for cases when more directed searches did not yield results. Datasets that have been assigned a digital object identifier (DOI) have, in most cases, been assigned a DOI through the DataCite Registration Agency and are thus searchable via that interface. At this time, an openly accessible, consolidated registry for locating datasets across domains and repository type (for example, federal data centers, institutional repositories and standalone repositories like Dryad or figshare) does not exist. There are repository registries, such as Re3data.org, that facilitate locating a data repository of interest, but there isn’t a mechanism to search across the repositories that are listed therein. We did not target specific databases or repositories unless they were explicitly mentioned in the DMP because it would be too time-intensive to search every known database by hand.

### NSF Funded Journal Articles Data and Protocol

We used the Thomson Reuters Web of Science database to identify journal articles produced by OSU faculty and funded by the National Science Foundation in the years 2012, 2013, and 2014. We selected this year range to attempt to minimize the number of papers that were affiliated with NSF funded projects from before the NSF data management plan mandate went into effect in 2011. This query to Web of Science resulted in 1013 journal articles for which we exported all of the data allowed by the database including, but not limited to, authors, title, journal title, and DOI. From this list of journal articles, we selected a random sample of 120 articles to review.

We reviewed each article to determine whether data were shared with the article using the following steps:

1. Scan the landing page for the article at the journal website for links or references to shared or supplementary data
2. Scan the article for information about shared data in the acknowledgments, supplementary data, appendices, and references
3. Scan the methods section for links or references to the datasets used in the paper (including links to repositories, accession numbers, references to data sources, etc.)
4. Search the entire document for the word “data” and scrutinize all mentions of the word for references to shared data

If the paper is related in some way to simulation or modeling, search the entire document for the words (and variants thereof) “parameter”, “calibration”, and “validation” and scrutinize all mentions of these words for references to shared data.

### Data Sharing Evaluation Protocol

Evaluations for each element of DATA (Discoverable, Accessible, Transparent, and Actionable) were made for each resource (project or journal article; see below for evaluation protocols) to assess the quality of data sharing for each source. For each DATA element, the resource was assigned a score of insufficient (0), partial (1), or full (2) compliance with the element of data sharing. A final DATA score was assigned by summing the scores from individual DATA elements. We assessed the data sharing practices for journal articles and funded projects based on the criteria in Table 1.

**Table 1:** Scoring criteria for the effectiveness of data sharing.

Discoverable		
*Score*	***Journal Article Scoring Criteria***	***Project Scoring Criteria***
0	No link or indication of data source in the data from the paper OR non-actionable mention of data location (e.g. broken links, mention of source without link)	Data cannot be found OR non-actionable mention of data location (e.g. broken links, mention of database but data in database cannot be directly linked to project)
1	A reference to the location or source of the data but no specific indication of the data used (e.g. a link to an external database) OR data shared are not all of the data used in the paper	The data can be found at the researcher's home page, a research group page or a project web page OR data shared are not all of the data used in the project
2	A direct link and/or persistent identifier for the dataset AND data are complete	The dataset can be found via a search at a subject repository or regional or national network, Google, or the DataCite Metadata Search AND data are complete
Accessible		
*Score*	***Journal Article & Project Scoring Criteria***	
0	Data shared through a closed or subscription access platform or accessed by request	
1	Data shared through a platform that requires some barrier to access such as a requirement to obtain permission to use the data OR data is shared through a closed-access source (e.g. journal, repository)	
2	Data shared in an open repository or platform or source (e.g. OA Journal, open repository, etc.)	
Transparent		
*Score*	***Journal Article & Project Scoring Criteria***	
0	No documentation provided for the data	
1	Some documentation provided for the data but lacks clear description of details such as how data were collected, analyzed or processed; description of units or headers; description of blanks; etc. Documentation may include a reference to the methods section of the paper	
2	Readme file, data dictionary, or other metadata shared with the dataset that provide clear details about the nature and content of the data	
Actionable		
*Score*	***Journal Article & Project Scoring Criteria***	
0	Data are not in a format that is usable in an analysis application (e.g. shared in a PDF or as a Fig)	
1	Data are in a format usable in an analysis application but are formatted in a way that makes use difficult (e.g. spreadsheets not in regular row-column form). OR data are shared in a proprietary or non-open format (e.g.xls,.doc,.mat,.sas,.shx)	
2	Data are in an open or non proprietary format (e.g.csv,.xlsx,.txt, etc.) with usable formatting	

## Results

### NSF Funded Projects

We reviewed thirty-three NSF data management plans acquired from the OSU Office of Sponsored Programs and attempted to locate shared data resulting from the associated projects. Of these, eight DMPs (24%) were associated with proposals for workshops, equipment, conferences or other activities that were not expected to generate data. That left 25 NSF-funded projects with DMPs available for which shared data could potentially be found [17].

**Fig 1:**
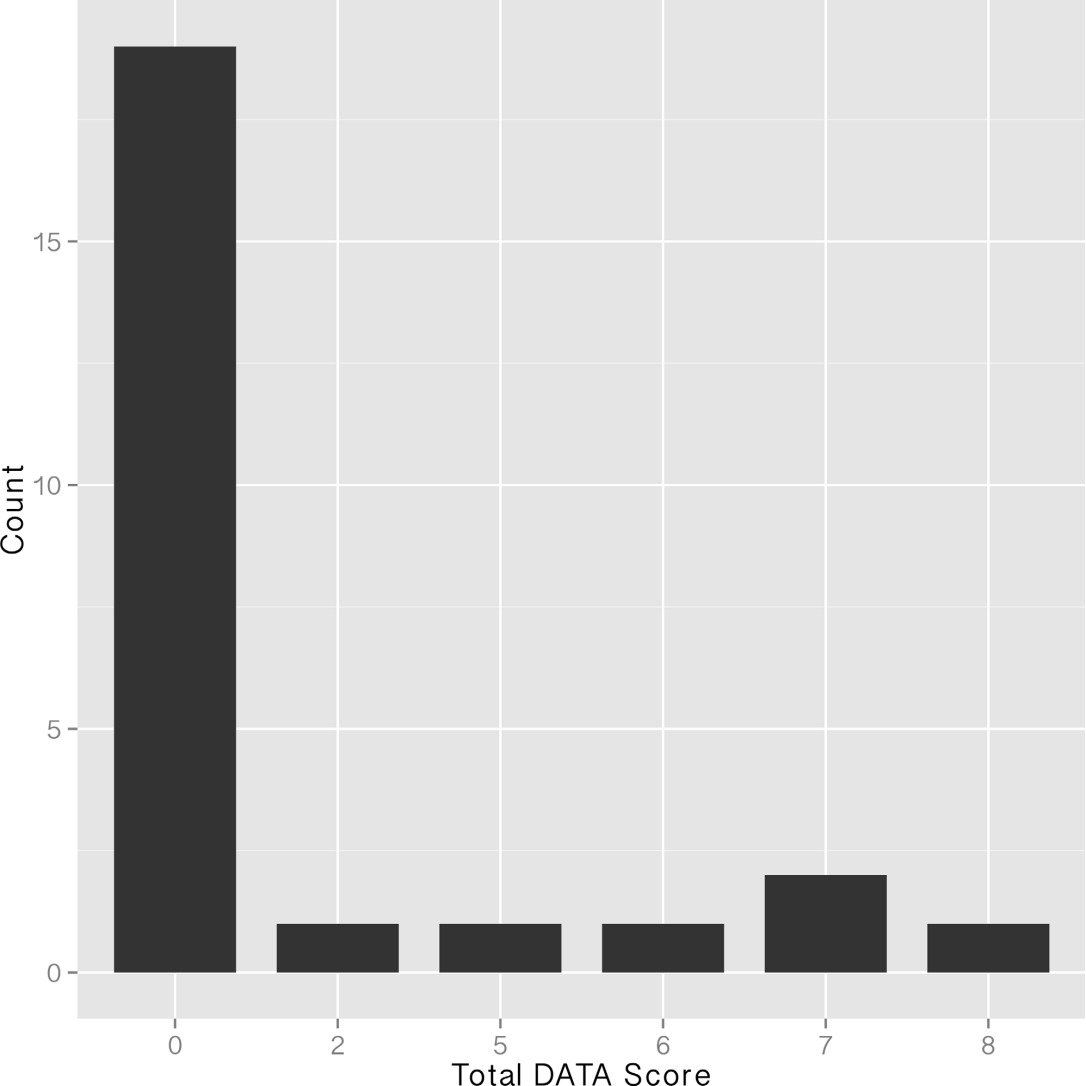
Total DATA scores from 25 NSF-funded projects, as located via data management plans

Of the 25 projects for which we attempted to locate shared datasets and generate a DATA score, nineteen (76%) had an overall score of 0 (Fig 1). Of the remaining six projects, one each had a score of 2, 5, 6 or 8, and two had a score of 7 (Table 2).

**Table 2:** Non-zero DATA scores from 25 NSF funded projects, with element scores shown.

DATA Score	Discoverable	Accessible	Transparent	Actionable
2	2	0	0	0
5	2	2	1	0
6	1	2	2	1
7	1	2	2	2
7	1	2	2	2
8	2	2	2	2

**Fig 2:**
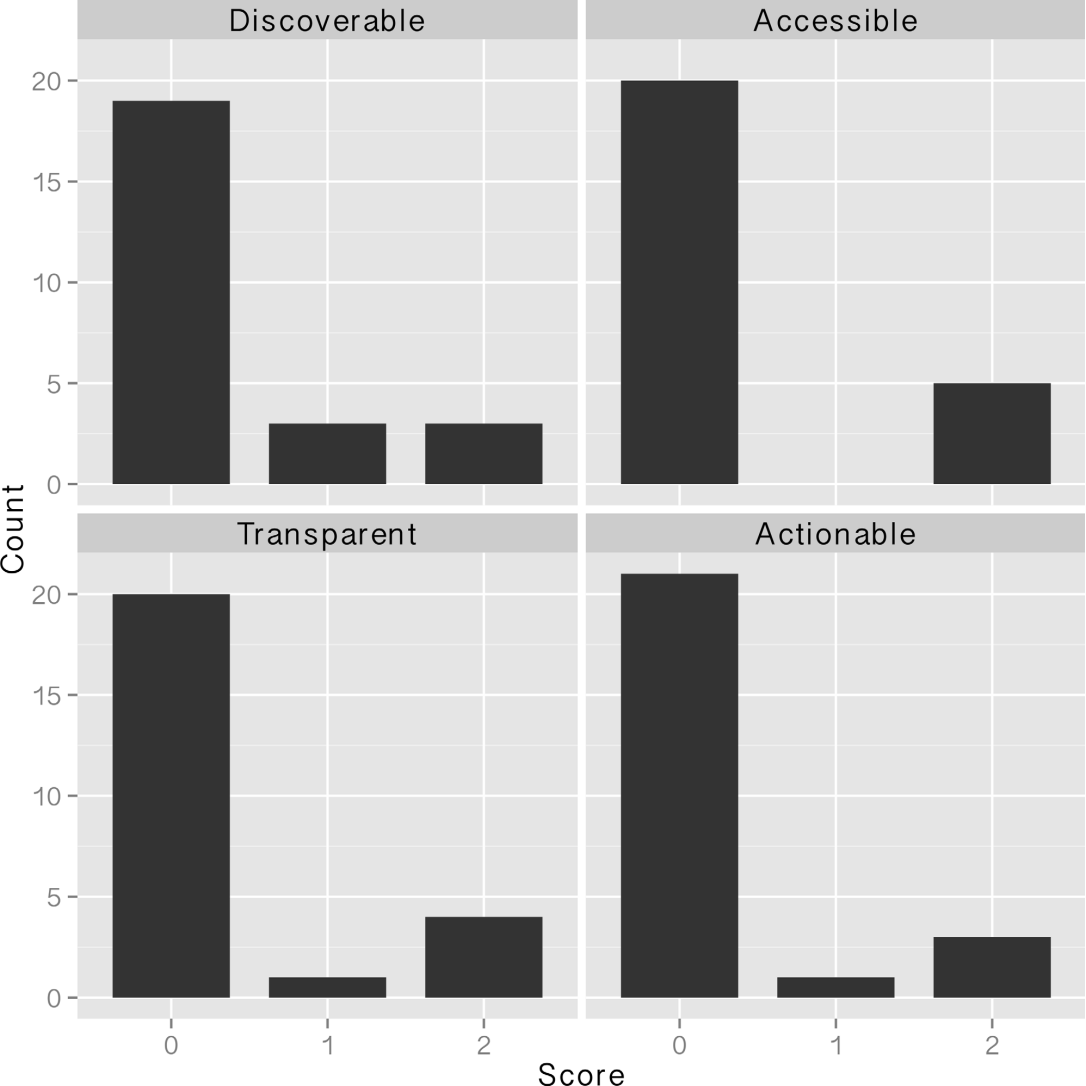
Element-wise DATA scores from 25 NSF-funded projects, as located via data management plans.

Element-wise DATA scores, which range from 0 to 2, were mostly clustered at either 0 or 2 (Fig 2). The exceptions were two projects that scored a 1 for Discoverable, and two other projects that had an element score of 1 for either Transparent or Actionable (Table 2; Fig 2). Three projects scored a perfect score (2) across the ‘ATA’ categories of Accessible, Transparent, and Actionable. Of these three projects, one further scored a 2 for Discoverable (for a perfect DATA score of 8), while the other two scored a 1 in the Discoverable category. One project scored a 1 in Actionable for using a deprecated file format (.xls). The project with a DATA score of 5 was rated a 2 for Discoverable and Accessible, a 1 for Transparent (incomplete metadata), and 0 for Actionable (zipped files failed to open). The remaining non-zero DATA score was rated as a 2 for Discoverable and 0s for all other categories because the data and documentation were on “proprietary hold” (embargoed) and could not be accessed.

### NSF Funded Journal Articles

We reviewed 123 research papers out of the 1013 retrieved from WOS, or about 12% of the articles. Of these, 19 were not analyzed either because our institution does not have subscription access to the journal in question (n = 7) or because the content of the article did not include data (e.g. it was entirely computational, theoretical, or mathematical; n = 12).

**Fig 3:**
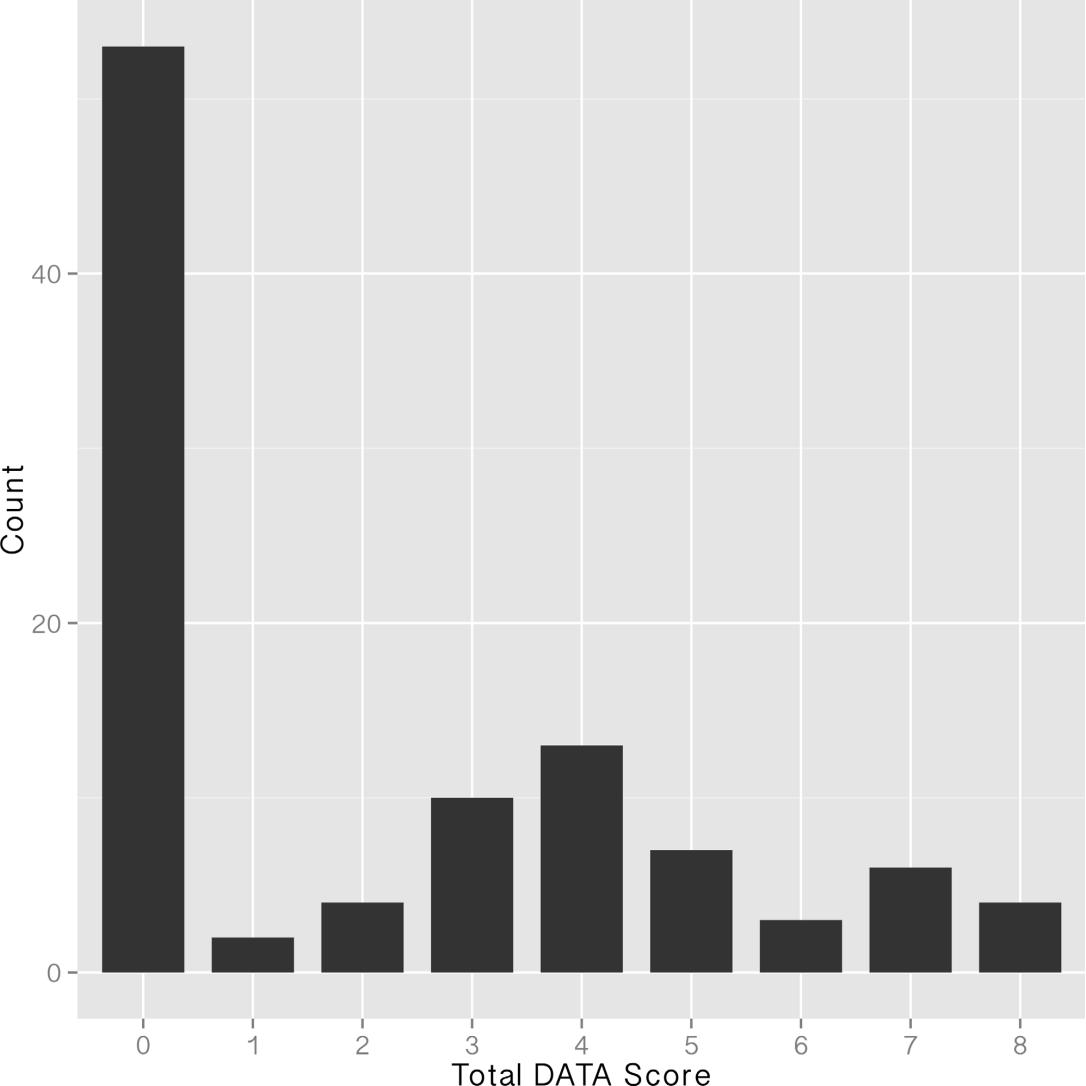
Total DATA scores from 104 NSF-funded projects, as located via journal articles.

DATA scores for the 104 articles that we scored ranged from 0 (the minimum score) to 8 (the maximum score), though the distribution of scores was heavily skewed towards low values with 55 journal articles scoring zero (Fig 3). Non-zero scores were primarily clustered in the 3–5 range (61% of non-zero scores) while only 20% or non-zero scores were in the 7–8 range. This peak around DATA scores from 3–5 is largely explained by the distributions of individual DATA score elements.

**Fig 4:**
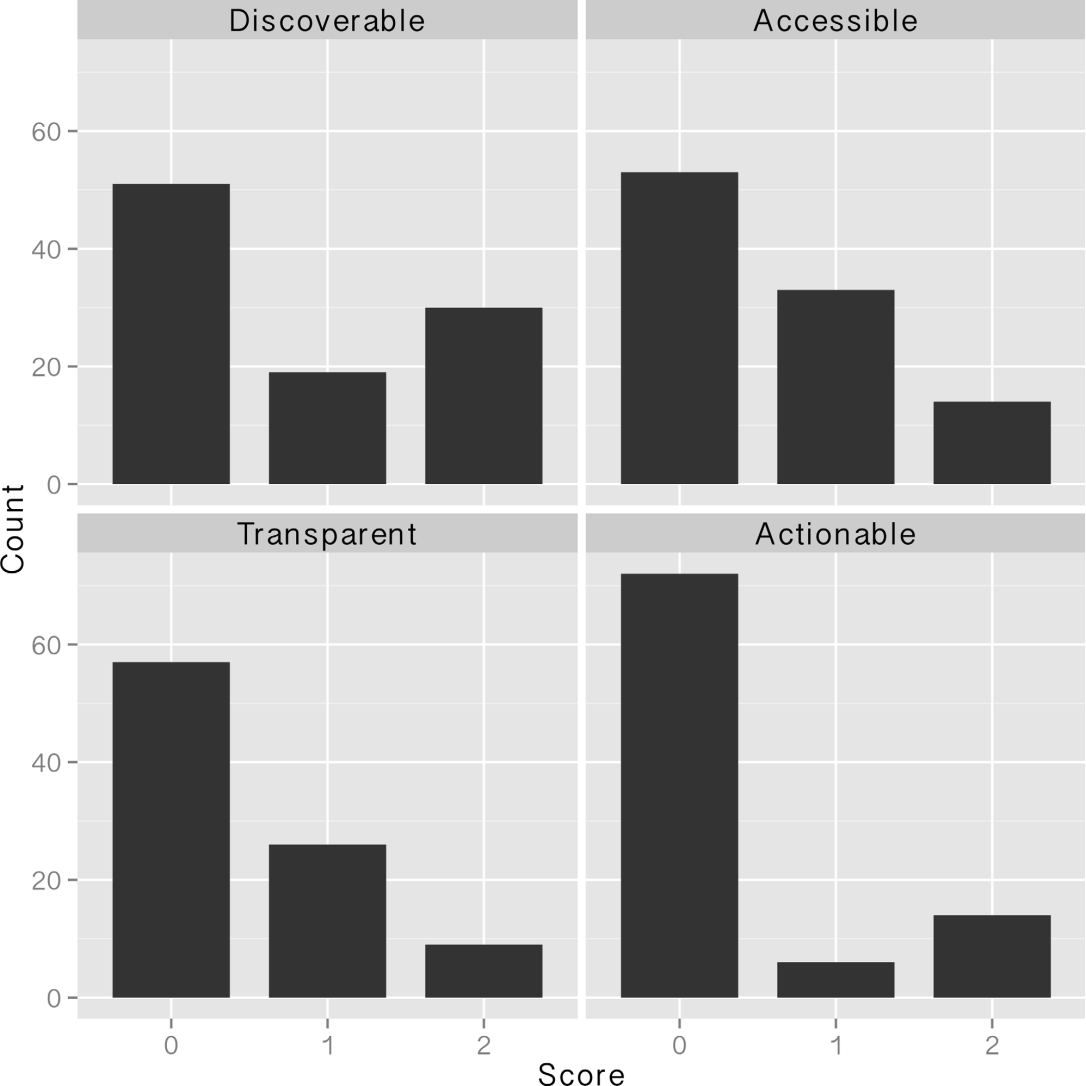
Element-wise DATA scores from 104 NSF-funded projects, as located via journal articles.

Element-wise DATA scores were primarily clustered around zero, as expected given the overall DATA score distribution, with slightly more than half of Discoverable and Accessible scores and about 60% of Transparent and Actionable scores ranking zero (Fig 4). Non-zero Discoverability and Accessibility scores were mirrored, with more Discoverability scores ranking 2 than 1 and more Accessibility scores ranking 1 than 2. Transparency and Actionability scores were similarly ranked with relatively few 2 scores compared with 1 scores.

## Discussion

In part, the differences seen across DATA element scores in journal articles are due to the fact that when data are not discoverable, the subsequent DATA elements cannot be evaluated, resulting in low scores for those elements. For example, data may be linked directly from the journal article, resulting in a high Discoverability score, but the data may reside in a closed-access repository (low Accessibility), contain little or no metadata or documentation (low or zero Transparency) and be formatted in such a way that makes use difficult, such as a proprietary data format (low or zero Actionability score).

Similarly, it is helpful to explore, for high DATA scores (6 and 7) that are not perfect (8), what DATA elements prevented the sharing from scoring higher. Of the nine articles with a DATA Score of 6 or 7, 6 had insufficient (score of 1) Accessibility scores due to the data being shared in a closed access venue. Insufficient Transparency for these articles was due to poor documentation that either was vague or relied on scrutiny of the methods section of the article to understand the data. Insufficient Discoverability was largely due to links to the data not leading directly to the dataset, rather the links led to external databases that required further action to find and acquire the dataset. Somewhat surprisingly, none of these high scoring datasets had Actionability scores less than 2.

Unlike the broad distribution that we observed in the DATA scores associated with journal articles (Fig 3), which indicates variable levels of data sharing effectiveness, we found that project-level datasets were either not shared (DATA = 0; 76% of projects) or were shared fairly effectively (Fig 1). Project level datasets that garnered DATA scores from 6–8 (N = 4) have characteristics that qualitatively inform our understanding of factors that are associated with effective data sharing for project data. In three cases the data were shared at a disciplinary (N = 2) or general (N = 1) data repository. This finding indicates that existing infrastructure likely facilitates data sharing, both directly, by providing a platform, and indirectly, by reinforcing a cultural norm around data sharing.

Despite our relatively small sample size, our results clearly demonstrate that the creation of a data management plan has very little bearing on whether or how datasets are shared. Principle investigators (PIs) often proposed to share data in several locations, in many cases for reasons that were not driven by the data itself (as an example, a justifiable case would be depositing different data types into separate, specialized repositories). In one case of sharing locale not being driven by the data, a PI indicated that they would share the same data both on a project website and via a domain repository. From a practical standpoint, such an approach to data sharing creates extra work for the PI, introduces potential issues for version control and authority, and makes it challenging to properly cite the dataset. Other DMPs proposed to share data in very limited or suboptimal ways, again for reasons that we couldn't associate with characteristics of the data. In some cases, such as for large datasets that don’t have a well-supported data center, sharing from peer-to-peer by request can be justified. In the case of the four DMPs that proposed sharing by request, however, we could not identify such justification. Four DMPs stated that they would share data via conference presentations. Ten DMPs stated that data would be shared via a PI or project website, but data were only discovered in these locations in two cases. Personal and institutional websites are not an ideal option for sharing data [13], mostly because access to websites and the websites themselves are not persistent. URLs often change over time, and websites may be deleted upon faculty relocation or for lack of funding. The ubiquity of this option among our sample of DMPs may indicate a need for more guidance from funding agencies and data service providers to facilitate the adoption of more robust options for data sharing.

The NSF does not currently require datasets to be shared with corresponding journal articles, though there is an impending expectation that the datasets that underlie papers will be shared after the OSTP mandates are fully realized [18]. That said, in many ways the journal article is one of the easiest and clearest venues for data sharing in the sciences, and data sharing for journal articles helps ensure at least a minimal level replicability or validation for projects (e.g. [19,20]). Journal articles represent a finite and fixed set of data when compared with the data for a whole project, and there is evidence that many researchers find journals to be preferred venues for data sharing [16]. In fact, we see much worse levels of sharing at the project level than at the paper level, perhaps because of the boundaries established by the paper as a discrete “package” of data to share. It is possible that for a researcher who is new to data sharing or reluctant to do so, deciding which parts of an entire project dataset should or could be shared would be a more difficult or intimidating problem than sharing the smaller set of data that underlies a paper.

Our protocols for finding shared data in articles and for projects may seem overwrought, but are indicative of a lack of consistency, for both data sharers and data sharing venues (including journals), about how data should be referred to and cited. When searching journal articles, data were almost never shared as a citation and were often found in acknowledgments, mentioned or linked in methods sections (but not formally cited), and occasionally referred to elsewhere in the document. When data existed in an external repository, authors often provided a link to the repository itself, rather than to the record for the dataset in the repository, even when persistent identifiers/links were provided for said datasets. It seems clear that there is some confusion about how data should be treated when referenced in the academic literature. This confusion creates a problem for identifying shared data associated with journals, not to mention problems with formal attribution and data being treated as a first-class scholarly output [21]. Researchers and publishers can play a role in ensuring proper citation of datasets by following community developed best practices for data citation, such as the Joint Declaration of Data Citation Principles produced by FORCE11 [22].

There are a handful of shortcomings to our methods that are worth noting. First, the data that we collect from Web of Science for the NSF projects database are not entirely representative of the body of research conducted at our university or in academia as a whole. While the content of Web of Science represents a very large body of journals, it is unclear the extent to which it represents a complete or representative view of the research being conducted at OSU and thus a complete view of data sharing practices associated with academic journal articles. We also have some concerns about the extent to which Web of Science has accurately represented the organizational and funding agency affiliations associated with journal content, though it is unclear in what direction (more or fewer journal articles) these accuracy concerns would move the data used in this study. That said, research at OSU is heavily focused in science, technology, engineering and math (STEM) fields and the funding agency we are examining (the National Science Foundation) is likewise STEM focused, so one might expect Web of Science content to represent a robust, if not a complete, body of research. Our assumptions about article publication dates (2012–2014) representing output from projects funded after the NSF data management plan policy went into effect may be somewhat flawed given the potential for many-year lags in production of scholarly articles from research funding. Last, our methods for identifying what data were used in a research article may be subject to our lack of expertise in the subject areas at hand. For instance, it is possible that the data and/or methods implicitly describe the data and the venue in which it is shared. However, this type of implicit sharing and documentation is problematic according to a number of our DATA criteria and, we believe, subject expertise should, in most cases, not be a requirement for identifying data sources in a research article.

There are also some potential limitations in our approach to locating datasets using information contained in data management plans. The DMP requirement went into effect four and a half years prior to the start of this research project, but fifteen of the twenty-five projects for which we had DMPs had project end dates that are less than one year from the time of this investigation. In spite of this however, seven out of the eight projects that had non-zero DATA scores had project end dates within the last year. This indicates that, in our dataset, recently completed projects were not any less likely to have shared data than projects that ended a year or more ago. Another potential limitation in our approach is the fact that we are not domain experts in most of the fields represented in our data. Some DMPs pointed in a general way toward specific repositories or metadata registries, and we may not have had the domain expertise necessary to locate the data in the repository. In cases where a DMP identified a repository for sharing and we could not locate data there, it’s possible that we were simply unable to locate the data when someone more familiar with the database could have. Last, it’s not clear from our small sample of DMPs the extent to which the mechanism that researchers propose to use for sharing adequately reflects their eventual data-sharing behavior. In two cases we located data in places that were not indicated in the DMP (versus six cases where the dataset was where we expected to find it). More research is needed on how well data management plans actually reflect the data management and sharing behaviors of the researchers who create them.

When tracking down datasets for projects or for journal articles, it may be difficult for a non-expert, or even an expert, to identify whether the entire dataset or only a portion of the dataset has been shared. This is not a new problem, and given the lack of effective data sharing that we observed, it’s not clear whether sharing mandates, the availability of infrastructure, or other factors are having any effect on the quality or completeness of shared data. There have been some discussions in the literature about how to verify the veracity of a dataset (e.g. the Scientifically Unique Identifier), [21] but the details of how to implement these systems are limited. An important area of investigation for all data sharing communities will be identifying which factors positively affect data sharing behaviors over time.

Finally, it would be useful, though out of scope for this study, to explore how data sharing for projects and publications from non-STEM organizations and agencies differs from what we see as part of this project.

## Recommendations

Authors and data producers who are seeking to share data can consider the DATA score as a starting point for understanding whether their intended methods for data sharing meet basic standards for data sharing. It is also important for authors to consider other data sharing best practices available through the academic literature, including papers by White et al. [13], Schofield et al. [1], and Kervin et al. [14], and to pay attention to any community standards that are or have emerged from their communities of practice. We would like to stress that while there exist important and emerging standards for data sharing (e.g., [15]), these standards can present barriers to compliance for researchers. Specifically, the focus of some standards (including the FAIR Data Guiding Principles) on machine readability, automation, and the use of appropriate ontologies for interoperability present barriers to researchers in domains where these modes of sharing are not the norm. That said, our DATA rubric calls for open data sharing, which may create barriers to achieving high DATA scores in cases where data cannot be shared openly. While sharing data in a manner that achieves a high DATA score almost certainly does not meet the high standards set elsewhere, we feel that the standard set by DATA is achievable for the majority of researchers and goes a long way toward providing usable shared research data. It is important, however, to acknowledge that a perfect DATA score is not always a useful or achievable goal. There exist well-known use-cases for restricting access to datasets (e.g. proprietary information, personally identifiable information), and it is important that users of the DATA rubric apply it in the context of the project in question, where possible.

What follows is a handful of other recommendations for authors and data producers that emerge from examining data sharing for projects and via journal articles. First, citation of datasets should be treated much the same as citation of journal articles, i.e. cite the dataset (including a unique identifier, if extant; [23], not the database, and be sure to cite the data in your references. Add the citations to your datasets in the same places where you would list your publications, e.g. on your curriculum vita and profile or research group web page, and on your NSF biosketch. One of the biggest Discoverability problems we saw with data sharing through journal articles was that citation styles and methods varied widely, making it difficult to find data that was meant to be shared. Others have covered ground before on how and why to properly cite data [8,9,23], and we will not cover that ground again here. Second, consider sharing your data through an open repository or journal. Many of the Accessibility issues we experienced when trying to find data for this project were related to the content being shared through a closed access journal or repository. There is a large and growing number of open repositories available for deposit of datasets, ranging from disciplinary to general purpose to institutional repositories. We recognize, as do many others, that not all data are appropriate for, or can find a home in, a disciplinary repository and we encourage researchers to consider the variety of general purpose or institutional repositories available for use (see www.re3data.org for examples). Last, we recognize that data sharing and meeting even the minimum standards for sharing we have set out here with DATA can be difficult and time consuming, and we encourage authors to engage with research data service providers either at the local level (often part of their academic library) or at the national level (e.g. DataONE - www.dataone.org, DataQ—researchdataq.org, etc.) for assistance and advice.

Our recommendations for publishers are threefold. First, set a policy for data sharing. There is plenty of guidance in the literature to facilitate the development of strong data sharing policies (see [6,21]), and it is clear from the work presented by Ferguson [16] and the work in this paper, that researchers are interested in sharing data alongside their publications. Second, guidelines for how to identify what data were used in the research (including data citation guidance), and possibly going as far as requiring proper declaration of what data were used in the research, would lead to a great deal of clarity for those seeking to reproduce the work or to create novel science with the data used in the work. Last, when data are shared through a journal article, even if the data are not shared via the journal (i.e. if they are shared through an external repository), it is important for the journal to take some responsibility for ensuring the quality of the shared data. How this process happens, whether by editorial or peer review or some other process, is up to the publisher, but it is crucial for journals not to wash their hands of responsibility for the quality of the shared data that support their articles. Data shared inadequately as part of a journal article reflects poorly on both the researcher and the journal.

As with publishers, we recommend that data repositories take some responsibility for ensuring the quality of the data and documentation deposited therein. Specifically, consider the Transparency and Actionability elements of our DATA definition of data sharing: does the dataset deposited have sufficient metadata, and are the data in a format that is actionable and open? While the repository manager(s) cannot always know the domain-specific details for a dataset, they may be able to require some minimum standards for characteristics of data shared in their repositories.

Last, our recommendations to funding agencies are aimed at encouraging them to engage more fully with grantees, journals, repositories, and data services providers to help ensure robust and practical standards for data sharing. Currently many federal funding agencies, in responses to the OSTP Memo of 2013 [5], have backed away from setting specific requirements for data sharing, instead calling on “communities of practice” to set these standards. It is with noble intent that the agencies have refused to lay down best practices from “on-high”, and to set multi-year timelines for planning to provide best-practices for data sharing. Unfortunately, the result that we see in this study, and that others have seen [1,24], is an environment of confusion and low-quality shared data. We believe that it is possible to establish definitions for minimally useable shared data, as we have done in this paper, without being overly prescriptive and still allowing for communities of practice to evolve.

## Conclusions

It is paramount to keep in mind the purpose of data sharing, which is to make the data functional for reuse, validation, meta-analysis, and replication of research, and that policies put in place regarding data sharing should keep those goals in mind. We found that the effectiveness of data sharing by federally funded researchers was poor in the large majority of cases. The content of data management plans revealed that researchers are largely unaware of how or where to share their data effectively. In several cases, even when a DMP did describe an appropriate data sharing strategy, we could not locate the data where it was proposed to have been. Attempts to locate the datasets associated with published articles indicated that researchers need more support and instruction in how, where and when to cite their own datasets. Researchers also need more support in the development of effective descriptive and structural metadata to support their shared data. We have made simple recommendations to data producers, publishers, repositories, and funding agencies that we believe will support more effective data sharing. Ultimately, as data sharing requirements are quickly becoming more common across funders, and will eventually be enforced, it is time for funding agencies to engage meaningfully in setting minimum definitions and expectations for sharing. Identifying a minimum definition of sharing in a broad but meaningful way would provide much-needed structure for researchers while still leaving room for domain-specific tuning to meet current and emerging domain best practices.

## References

[1] Schofield PN, Bubela T, Weaver T, Portilla L, Brown SD, Hancock JM, et al Post-publication sharing of data and tools. Nature. 2009;461: 171–173. 10.1038/461171a 19741686 PMC6711854

[2] National Science Foundation. Significant Changes to the GPG. National Science Foundation; 2011 Available: http://www.nsf.gov/pubs/policydocs/pappguide/nsf11001/gpg_sigchanges.jsp

[3] National Science Foundation. Part II, the NSF Award and Administration Guide (AAG). Proposal & Award Policies & Procedures Guide. Arlington, Virginia, USA: National Science Foundation; 2013 p. Section D.4.b. Available: http://www.nsf.gov/pubs/policydocs/pappguide/nsf13001/aag_6.jsp#VID4

[4] National Science Foundation. NSF 95–27 Grant Proposal Guide. Arlington, Virginia, USA: National Science Foundation; 1995 Aug p. 47. Report No.: NSF 95–27. Available: http://www.nsf.gov/publications/pub_summ.jsp?ods_key=nsf9527&org=NSF

[5] Holdren JP. Memorandum for the heads of executive departments and agencies: Expanding public access to the results of federally funded research Executive Office of the President, Office of Science and Technology Policy; 2013 Available: http://www.whitehouse.gov/sites/default/files/microsites/ostp/ostp_public_access_memo_2013.pdf

[6] Sturges P, Bamkin M, Anders JHS, Hubbard B, Hussain A, Heeley M. Research data sharing: Developing a stakeholder-driven model for journal policies. J Assoc Inf Sci Technol. 2015; n/a–n/a. 10.1002/asi.23336

[7] The Editorial Board. Scientists Who Cheat. The New York Times. 1 Jun 2015. Available: http://www.nytimes.com/2015/06/01/opinion/scientists-who-cheat.html. Accessed 1 Jun 2015.

[8] Martone ME. Brain and Behavior: we want you to share your data. Brain Behav. 2014;4: 1–3. 10.1002/brb3.192 24653948 PMC3937699

[9] Kratz J, Strasser C. Data publication consensus and controversies. F1000Research. 2014; 10.12688/f1000research.3979.1 PMC4097345 25075301

[10] McNutt M. Data, eternal. Science. 2015;347: 7–7. 10.1126/science.aaa5057 25554763

[11] Bloom T. Data Access for the Open Access Literature: PLOS’s Data Policy. Public Library of Science; 2013 Available: https://www.plos.org/data-access-for-the-open-access-literature-ploss-data-policy/

[12] National Science Foundation. Data Management & Sharing Frequently Asked Questions. In: National Science Foundation Website. 30 Nov 2010. Available: http://www.nsf.gov/bfa/dias/policy/dmpfaqs.jsp. Accessed 11 July 2015.

[13] White EP, Baldridge E, Brym ZT, Locey KJ, McGlinn DJ, Supp SR. Nine simple ways to make it easier to (re)use your data. Ideas Ecol Evol. 2013;6 10.4033/iee.v6i2.4608

[14] Kervin K, Michener W, Cook R. Common Errors in Ecological Data Sharing. J EScience Librariansh. 2013;2. doi:10.7191/jeslib.2013.1024

[15] The FAIR data Guiding Principles. In: Force11. Available: https://www.force11.org/group/fairgroup/fairprinciples. Accessed 10 July 2015.

[16] Ferguson L. How and why researchers share data (and why they don’t). In: Exchanges. 3 Nov 2014. Available: http://exchanges.wiley.com/blog/2014/11/03/how-and-why-researchers-share-data-and-why-they-dont/. Accessed 7 July 2015.

[17] Van Tuyl S, Whitmire AL. Data from: Water, water everywhere: Defining and assessing data sharing in academia Oregon State University Libraries; 2015 Available: 10.7267/N9W66HPQ PMC4757565 26886581

[18] National Science Foundation. Public Access Plan: Today’s Data, Tomorrow’s Discoveries: Increasing Access to the Results of Research Funded by the National Science Foundation. Arlington, Virginia, USA: National Science Foundation; 2015 Mar p. 31. Report No.: nsf15052. Available: https://www.nsf.gov/publications/pub_summ.jsp?ods_key=nsf15052

[19] Wicherts JM, Bakker M, Molenaar D. Willingness to Share Research Data Is Related to the Strength of the Evidence and the Quality of Reporting of Statistical Results. PLoS ONE. 2011;6: e26828 10.1371/journal.pone.0026828 22073203 PMC3206853

[20] Collberg C, Proebsting T, Moraila G, Shankaran A, Shi Z, Warren AM. Measuring Reproducibility in Computer Systems Research. University of Arizona; 2013 12 p. 37 Available: http://reproducibility.cs.arizona.edu/v2/RepeatabilityTR.pdf

[21] Lin J, Strasser C. Recommendations for the Role of Publishers in Access to Data. PLoS Biol. 2014;12: e1001975 10.1371/journal.pbio.1001975 25350642 PMC4211645

[22] Data Citation Synthesis Group. Joint Declaration of Data Citation Principles. San Diego, CA, USA: Force11; 2014 Available: https://www.force11.org/group/joint-declaration-data-citation-principles-final

[23] Ball A, Duke M. How to Cite Datasets and Link to Publications. DCC How-to Guides. Edinburgh: Digital Curation Centre; 2012 Available: http://www.dcc.ac.uk/resources/how-guides/cite-datasets

[24] Kim Y, Stanton JM. Institutional and individual factors affecting scientists’ data-sharing behaviors: A multilevel analysis. J Assoc Inf Sci Technol. 2015; n/a–n/a. 10.1002/asi.23424

